# Ectopic expression of BIRC5-targeting miR-101-3p overcomes bone marrow stroma-mediated drug resistance in multiple myeloma cells

**DOI:** 10.1186/s12885-019-6151-x

**Published:** 2019-10-21

**Authors:** Jahangir Abdi, Nasrin Rastgoo, Yan Chen, Guo An Chen, Hong Chang

**Affiliations:** 1 Dept. of Laboratory Hematology, Laboratory Medicine Program, Toronto General Hospital, University Health Network, 200 Elizabeth Street, 11E-413, Toronto, Ontario M5G 2C4 Canada; 2 Department of Hematology/Oncology, First Affiliated Hospital, Nanchang University, Nanchang, China; 3 Dept. of Laboratory Medicine & Pathobiology, University of Toronto, Toronto, Canada

**Keywords:** Bone marrow stroma, Multiple myeloma, Multi-drug resistance, miRNA, Survivin, Apoptosis

## Abstract

**Background:**

Multiple myeloma (MM) cells gain protection against drugs through interaction with bone marrow stromal cells (BMSCs). This form of resistance largely accounts for resistance to therapy in MM patients which warrants further exploration to identify more potential therapeutic targets.

**Methods:**

We performed miRNA/mRNA qPCR arrays and western blotting to analyze transcriptional and translational changes in MM cells co-cultured with BMSCs. Drug cytotoxicity and apoptosis in MM^GFP^-BMSC co-cultures were measured using fluorescence plate reader and flowcytometry, respectively. miRNA was overexpressed in MM cell lines using Lentiviral transduction, miRNA-3’UTR binding was examined using luciferase assay.

**Results:**

We found that BMSCs downregulated miR-101-3p and upregulated survivin (BIRC5) in MM cells. Survivin was downregulated by miR-101-3p overexpression and found to be a direct target of miR-101-3p using 3’UTR luciferase assay. Overexpression of survivin increased viability of MM cells in the presence of anti-myeloma drugs, and miR-101-3p inhibition by anti-miR against miR-101-3p upregulated survivin. Furthermore, overexpression of miR-101-3p or silencing of survivin triggered apoptosis in MM cells and sensitized them to anti-myeloma drugs in the presence of BMSCs overcoming the stroma-induced drug resistance.

**Conclusions:**

Our study demonstrates that BMSC-induced resistance to drugs is associated with survivin upregulation which is a direct target of miR-101-3p. This study also identifies miR-101-3p-survivin interaction as a druggable target involved in stroma-mediated drug resistance in MM and suggests it for developing more efficient therapeutic strategies.

## Background

Multiple myeloma (MM) remains incurable due to drug resistance which develops partly because MM cells are protected by bone marrow stromal cells (BMSCs) [1–3]. Such stroma-mediated protection of MM cells may work through soluble factors released from BMSCs or through cell-cell adhesion termed cell adhesion-mediated drug resistance (CAMDR) [4]. Thus, identification of potential druggable targets or regulatory mechanisms involved in this complex interaction warrants in-depth exploration.

miRNAs have prominent roles in MM pathogenesis [5, 6], and most miRNAs studied in MM have been found downregulated functioning as important tumor suppressors [5–7], such notion has also been evidenced by our lab publications [8–10]. Recent studies have provided important clues that aberrations of miRNA expression and function may contribute to drug resistance in MM [11–13], including microenvironment related therapy resistance [14, 15]. For instance, deregulated tumor suppressors miR-29b and miR-27a-5p in MM modulate anti-myeloma activity of bortezomib (BTZ) in MM cells by targeting the oncogenes SP1 and CDK5, respectively [16, 17].

The gene expression and function of MM cells may alter in the mutual direct (cell-cell adhesion) or indirect (release of soluble factors) interaction with BMSCs. It has been reported that IL-6 secreted from BMSCs may protect MM cells against BTZ-induced apoptosis by suppressing miR-15a in latter cells [18]. Furthermore, CAMDR of MM cells was abolished when miR-202 was overexpressed in BMSCs [19], and upregulation of miR-21 in MM cells following adhesion to BMSCs was reported to be associated with resistance to drugs [20]. High expression of some anti-apoptotic proteins, such as BCL2 family members, has also been suggested to contribute to stroma-mediated drug resistance in MM [21–23], especially MCL-1 which was demonstrated to be important in BMSC-mediated protection of MM cells against apoptosis [24]. The above findings indicate that inhibition of apoptosis in MM cells by BMSCs may partly underlie stroma-mediated drug resistance in MM. However; evidence to support at a mechanistic level the actual involvement of miRNA-target axis in drug resistance of MM cells interacting with BMSCs, are not convincing.

In the present research, we showed that BMSCs rendered MM cells resistant to drugs mostly through direct adhesion. Analysis of qPCR-based pathway-focused arrays in MM cells adhered to BMSCs vs MM cells cultured alone identified that BMSCs downregulated miR-101-3p and upregulated survivin (BIRC5) in MM cells even in the presence of BTZ. Downstream functional analyses also showed that targeting the miR-101-3p/survivin axis overcame stroma-induced drug resistance in MM cells.

## Methods

### Cells, cell culture

Human myeloma cell lines (HMCLs), RPMI-8226, U266, MM.1S, OPM2 and the immortalized human bone marrow stromal line HS-5 were obtained from ATCC; no further authentications were performed by the authors. HMCLs were maintained in RPMI medium containing 2 mM glutamine, supplemented with 10% FBS and intermittently with 100 U/ml penicillin and 100 μg/ml streptomycin. HS-5 cells were maintained in DMEM medium supplemented with 10% FBS and no antibiotics. Primary BMSCs from MM patients were isolated and expanded following a procedure explained before with some modifications [25]. Here we used freshly isolated bone marrow samples from newly diagnosed MM patients at our institute. The samples were applied to Ficoll-Hypaque density gradient centrifugation. Bone marrow mononuclear cells (BMMCs) were suspended in complete RPMI medium in small T-25 flasks for 2 h to let the adherent fraction adhere to plastic. Culture medium containing floating cells was removed and fresh complete DMEM was added. Medium was changed twice a week, this led to BMSCs confluency in about 3 weeks, which were used at passage 2. Malignant plasma cells in the floating fraction were isolated using magnetic beads according to our previous protocol [26] and used immediately for gene expression and functional experiments.

### Reagents and kits

Anti-myeloma drugs BTZ and carfilzomib (CFZ) were obtained from Cayman Chemicals (Ann Arbor, MI, USA), miScript miRNA PCR Array (MIHS-114ZA), miRNA cDNA synthesis (miScript RT II), mRNA isolation (RNeasy), miRNA isolation (miRNeasy) and Qiafilter plasmid Maxi kits were all from QIAGEN. For mRNA cDNA synthesis, Superscript III (Life Technologies) or iScript (Bio-Rad) were used. Immune-magnetic cell separation kit (EasySep, negative selection) was procured from Stem Cell Technologies. To perform gene (BIRC5) silencing, we used Ambion® *Silencer*® Select siRNA and Lipofectamine RNAiMAX transfection reagent (Life Technologies).

### MM^GFP^-BMSC co-culture model

To establish a co-culture model for studying drug response of MM cells in the presence of BMSCs, MM cell lines were transduced with GFP-expressing vectors and exposed to HS-5 or primary MM BMSCs. To this aim, GFP-expressing lentiviral particles were produced by packaging pGIPZ-GFP plasmid (empty backbone) into pMD2.G and psPAX2 vectors (Adgene plasmids, # 12259 and #12260, respectively, gifts from Didier Trono) using Lipofectamine 3000 in HEK-293T cell line. Forty eight hours post transfection, HEK293T supernatant containing lentiviral particles was harvested. HMCLs (5 × 10^6^) were transduced with viral particles (with an MOI close to 1.0) using spinocculation technique (polybrene 8.0 μg/ml, spinning at 2500 rpm for 1.5-2 h at 32 °C). Transduced cells were re-suspended in fresh pre-warmed RPMI medium plus FBS and kept for 2–3 days at 37 °C followed by maintaining in the presence of puromycin (2.5 μg/ml) to select for GFP-positive cells for further 3 days.

### GFP-based apoptosis and cytotoxicity assays

To perform apoptosis assay in co-cultures, GFP-tagged HMCLs cells were cultured in 3 settings: Seeded on BMSCs, separated from BMSCs using a transwell (TW) insert, or seeded on BMSCs pre-treated with Brefeldin-A (BFA), with or without BTZ or CFZ (5 nM). GFP-tagged HMCL mono-cultures were used as controls. Where required, BMSCs were pretreated with BFA (200 ng/ml) for 8 h, rinsed twice with warm PBS and used in co-culture with MM cells in RPMI medium. In latter medium BMSCs grew well during designated incubation times, consistent with a previous report on HS-5 co-culture with MM cells in RPMI medium for maximum 3 days [27]. After 48 h, percent apoptosis of gated GFP+ cells was determined using annexin-V/PI staining in flow cytometry. For cytotoxicity experiments, HMCLs in phenol red-free RPMI+FBS were seeded on BMSC-coated wells, after 6 h different concentrations of drugs were added. GFP fluorescent intensity was measured through a fluorescence plate reader (SpectraMax M5, Molecular Devices) after 72 h.

### mRNA and miRNA real time PCR arrays

Three GFP-tagged HMCLs, RPMI-8226, MM.1S and U266 were cultured with HS-5 cells or cultured alone for 24 h. MM cells from co-cultures were isolated using EasySep immunomagnetic bead assay yielding an almost 98% purity of MM cells (we also tried flow cytometric sorting of co-cultures by gating on GFP+ cells, however; cell viability was higher in magnetic assay, data not shown). Total RNAs including miRNAs were isolated from all samples and synthesized cDNAs were applied to amplification of 84 genes related to apoptosis, cell cycle and proliferation using qPCR. After normalization of all Ct values, fold regulations were calculated for cells adhered to HS-5 relative to cells in mono-cultures by using the 2^-∆∆Ct^ algorithm. In another experiment, the above culture scenario was applied to 84 miRNAs related to apoptosis pathway in HS-5-MM.1S co-culture relative to MM.1S monoculture after normalization of all Ct values to SNORD series (61, 68, 72, 95, 96A) of housekeeping miRNAs.

### Gene silencing and miR-101-3p inhibition

To knock down BIRC5 gene, 3 × 10^5^ cells from RPMI-8226 or MM.1S cell lines were cultured in a 24-well plate. Fifty picomole from siRNA or scrambled RNA was added in the presence of Lipofectamine RNAiMAX to each well to achieve a final oligo concentration of around 100 nM. Twenty four hours after transfection, samples were treated with 5 nM BTZ in the presence or absence of HS-5 cells and incubation extended to another 24 h followed by flow cytometric analysis of annexin-V/PI apoptosis or immunoblot detection of survivin protein. To inhibit miR-101-3p in RPMI-8226, cells were transfected with synthetic inhibitor of miR-101-3p (from GeneCopoeia) using HiperFect. We performed the transfection according to the HiperFect protocol of the company (Qiagen) for suspension cells.

### Lentiviral expression vectors

To produce lenti-GFP expression clones of miR-101-3p in HMCLs, we first packaged expression plasmids of miR-101-1 or scrambled miRNA into third generation packaging lentiviral vectors pRSV-Rev and pMDLg/pRRE (Addgene plasmids, #12253 and # 12251, respectively, gifts from Didier Trono [28]) in HEK-293 T cells. Then, RPMI-8226 and MM.1S cells (5 × 10^6^ cells each) were transduced with Lenti^GFP^-miR or Lenti^GFP^-NC viral particles using spinocculation technique as explained above. To overexpress BIRC5 gene in RPMI-8226 cells, we used the human BIRC5 ORF clone (EX-A3492-Lv122, GeneCopoeia) and its negative control vector (EX-NEG-Lv122). Briefly, 0.5 × 10^6^ cells per well were seeded in a 12-well plate and 1.0 μg of ORF clone or negative control in the presence of Lipofectamine 3000 was added to each well and incubated for 48 h. Cells were harvested to be assessed in MTT assay for drug response (5 nM BTZ or CFZ for 48 h) or to be applied to western blotting for measuring survivin protein level.

### Statistics

We used one- or two-way analysis of variance in GraphPad prism 6 software for data analysis in all experiments and *P* < 0.05 was considered significant.

### The following methods are included in the supplementary material

miRNA-target binding, luciferase assays and western blotting.

## Results

### BMSCs modulate a transcriptome in MM cells in the presence of BTZ

**Fig. 1:**
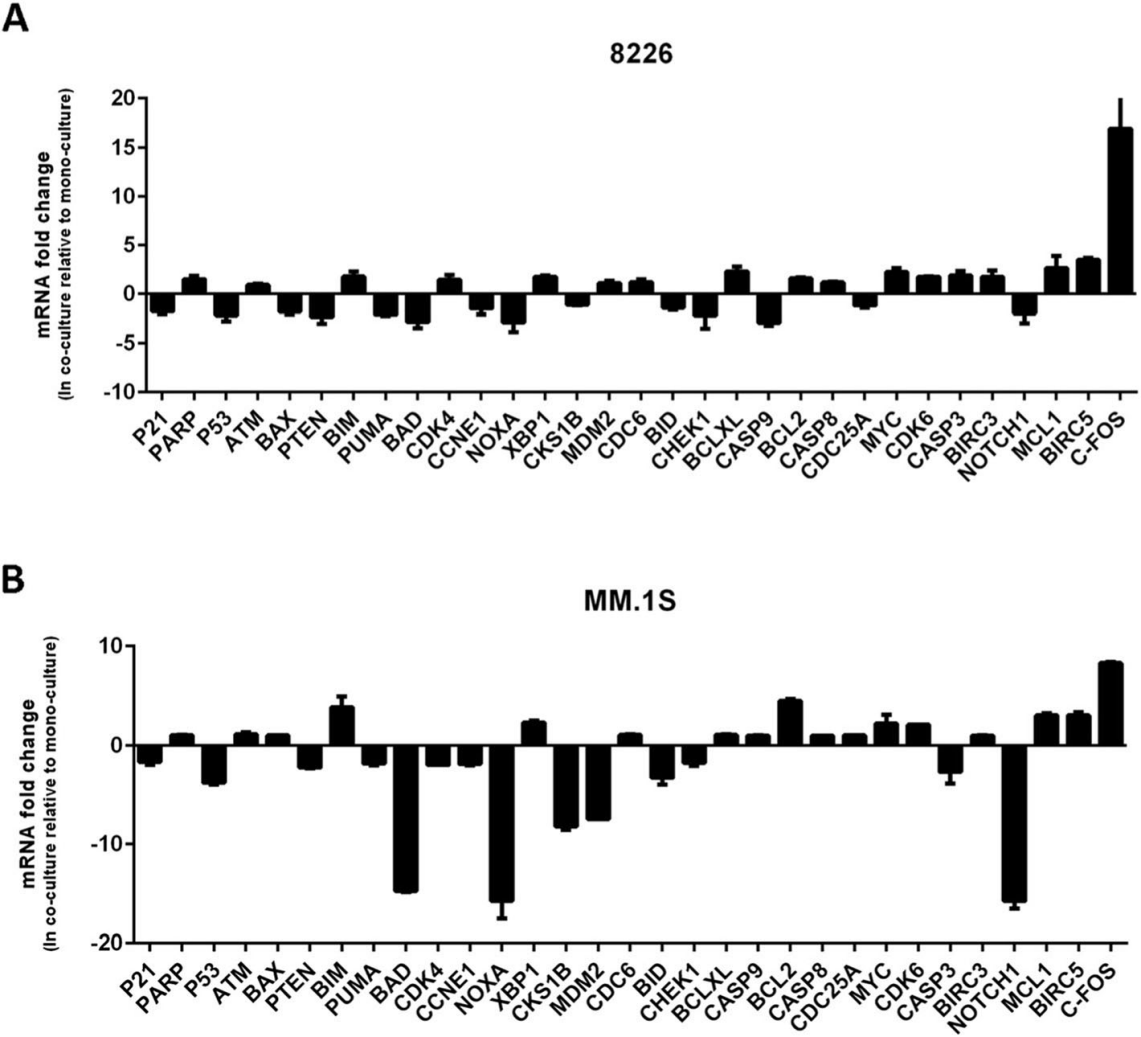
BMSCs modulate an array of mRNAs in MM cells. RPMI-8226, U266 and MM.1S cells were co-cultured with HS-5 cells in the presence of 5 nM BTZ for 24 h. MM cells were isolated using magnetic bead assay (EasySep), cDNAs were synthesized and applied to pathway-(apoptosis, proliferation, and survival)-focused mRNA qPCR array. The graphs for RPMI-8226 and MM.1S cells are the re-analysis of selected genes from an array of 84 (Additional file 1: Figure S1, clusterogram). The same graph for U266 cell line is in the supplementary (Additional file 2: Figure S2). The graphs show the fold changes of transcripts in MM cells co-cultured with HS-5 cells compared to MM cells cultured alone, details in the M&M

The pattern of gene expression in MM cells interacting with BMSCs in the presence of anti-myeloma drugs has not been explored. Elucidating this concept will help us to identify targets involved in stroma-mediated drug resistance. To address this issue, we co-cultured RPMI-8226, U266 and MM.1S cells with HS-5 cells in the presence of BTZ for 24 h and performed pathway-(apoptosis, proliferation, and survival)-focused mRNA qPCR array (Additional file 1: Figure S1, clusterogram). BTZ-treated human myeloma cell lines (HMCLs) cultured alone were used as controls. As recommended in such arrays, Ct values above 35.0 should be considered negative calls and ignored, also only fold regulations ≥2.0 or ≤ − 2.0 are taken into account for further analysis. With this approach, we selected 31 genes to re-analyze in qPCR. Out of these genes only *FOS* (immediate response), *BIRC5* and *MCL1* (anti-apoptotic), and *MYC* (cell proliferation) were confirmed to be consistently upregulated in the three cell lines by HS-5 cells (2–17 fold) (Fig. 1a,b, Additional file 2: Figure S2). However; for reasons explained below we focused only on survivin for downstream functional analysis.

### BMSCs upregulate survivin mRNA and protein in MM cells mainly through cell-cell adhesion even in the presence of BTZ

**Fig. 2:**
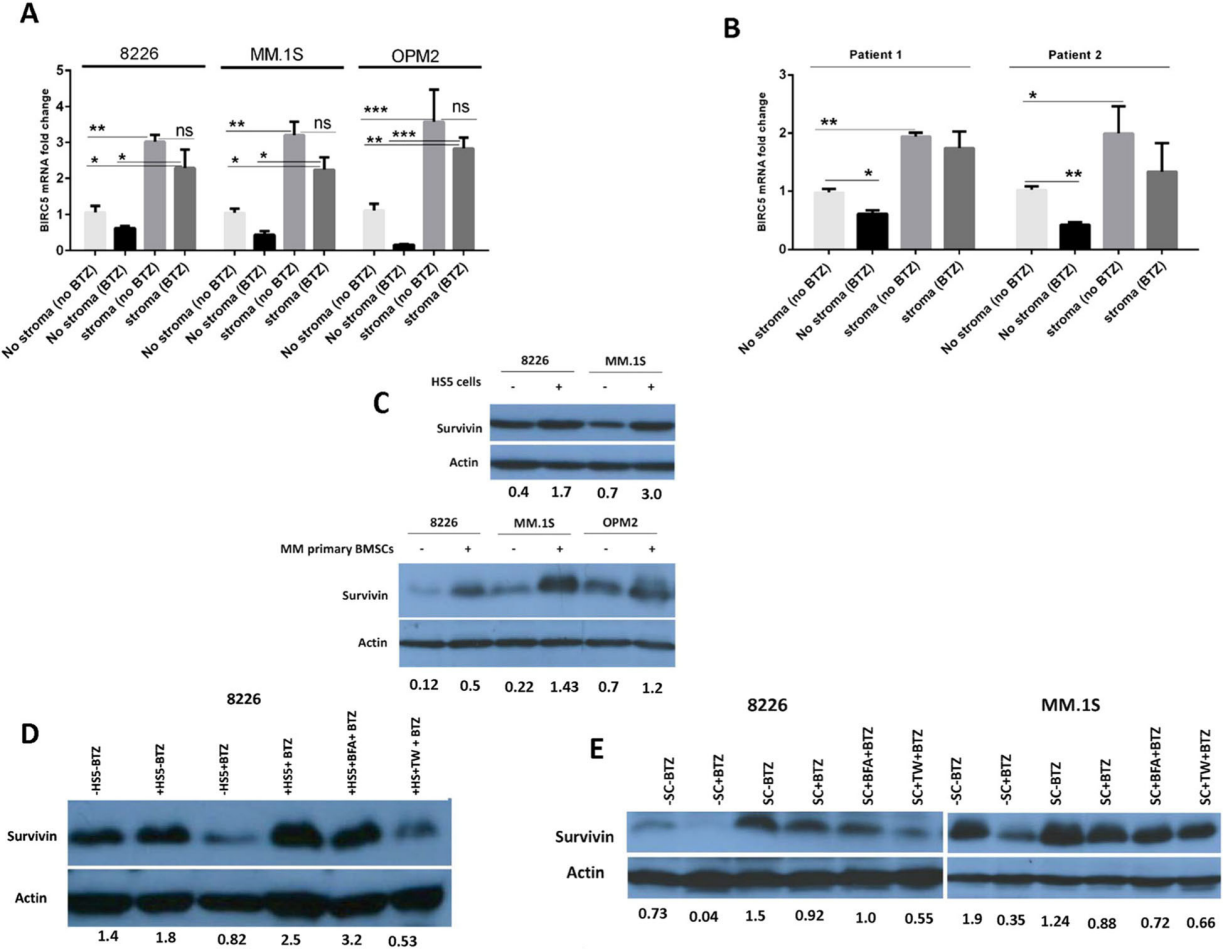
BIRC5 mRNA or survivin protein is upregulated by BMSCs in MM cells mainly through direct cell-cell adhesion even in the presence of BTZ. **a** MM cells were cultured for 24 h in the following settings: cultured alone with or without 5 nM BTZ, co-cultured with HS-5 cells with or without 5 nM BTZ, co-cultured with HS-5 cells pre-treated with BFA or separated with a TW insert. Cells from all conditions were harvested, cDNAs isolated and used in real time PCR for analysis of BIRC5 mRNA expression. Analyzed data are from three separate experiments, *p < 0.05, **p < 0.01, ***p < 0.001. **b** Primary malignant plasma cells from two MM patients were treated and analyzed as in **A** but in a 12 h incubation period. **c** MM cells were co-cultured with HS-5 cells or patient-derived BMSCs for 24 h in the absence of BTZ and survivin protein was measured using WB. MM cells cultured alone were used as controls. **d** RPMI-8226 cells were cultured for 24 h in the following settings: cultured alone with or without 5 nM BTZ, co-cultured with HS-5 cells with or without 5 nM BTZ, co-cultured with HS-5 cells pre-treated with BFA or separated with a TW insert. Cells from all conditions were harvested and applied to WB for measuring survivin protein. **e** The same scenario as explained in **D** was applied to RPMI-8226 and MM.1S cells but in the context of MM patient-derived BMSCs (SC). For densitometric quantification of WB bands, we used ImageJ software. The densities of survivin bands were divided by densities of their relevant actin bands and reported as shown

As mRNAs of *BIRC5*, *FOS*, *MCL1* and *MYC* were upregulated in HMCLs by HS-5 cells, we preferred to confirm this at protein level. c-FOS did not show a consistent stroma-mediated modulation at protein level among MM cells (data not shown). MCL-1 and c-MYC were moderately upregulated in RPMI-8226 cells by BMSCs but suppressed by BTZ in the presence or absence of BMSCs (Additional file 3: Figure S3**),** indicating that these proteins may not be involved in BMSC-mediated drug resistance. On the other hand, BIRC5 mRNA was partially suppressed by BTZ in the absence of BMSCs but upregulated when MM cells were co-cultured with BMSCs even in the presence of BTZ (Fig. 2a). The similar pattern was also observed with primary MM cells (Fig. 2b) further supporting the clinical relevance of these findings. In addition, analysis of the GEO dataset (GSE31159) form 13 MM patients’ BM samples revealed that co-culture of MM primary tumor cells with BMSCs tended to increase BIRC5 mRNA relative to MM cells cultured alone in 10 out of 13 patients (Additional file 4: Figure S4). Consistent with mRNA findings, HS-5 cells (Fig. 2c, upper panel) and primary MM BMSCs (Fig. 2c, lower panel) upregulated survivin protein in MM cells. Furthermore, survivin protein was downregulated by BTZ as reported in the literature before [29] but HS-5 cells (Fig. 2d) or MM patient’s primary BMSCs (Fig. 2e) upregulated it even in the presence of BTZ. In co-culture with BMSCs pre-treated with BFA, MM cells did not show any change in survivin protein in the presence of BTZ. However, when MM cells were separated from BMSCs by TW inserts, survivin protein was strikingly suppressed (Fig. 2d,e) suggesting possible involvement of survivin in stroma-induced drug resistance through direct cell-cell adhesion. BFA is an inhibitor of intracellular protein trafficking and was shown to effectively inhibit release of cytokines or exosomes from stromal cells [30, 31]. BFA effects may be reversible after removal of BFA, however, human MSCs incubated with 5 μg/ml BFA for 1 day could not restore IL-6 secretion for 72 h afterwards [32]. These findings imply that both soluble factors and direct cell-cell adhesion are involved in stroma-mediated modulation of survivin in MM cells, and that BMSCs upregulate survivin in MM cells irrespective of BTZ. Interestingly, these observations are in line with our GFP-based flow cytometry results for drug-induced apoptosis in stroma context. In the absence of stroma, RPMI-8226, MM.1S and U266 cells treated with BTZ or CFZ showed a high level of cell death, but it declined in the presence of HS-5 or MM primary BMSCs. Moreover, mean percent apoptosis for BFA vs TW conditions in RPMI-8226 and MM.1S cells in co-culture with HS-5 cells (Additional file 5: Figure S5A-C), respectively, was 45.07 vs 60.40 (*p* = 0.0035) and 55.20 vs 73.82 (*p* = 0.0284), however; in case of U266 cells it could not reach significance (57.50 vs 68.47, *p* = 0.089). Additionally, in all three cell lines the difference in apoptosis between TW and no stroma was not significant but it was for BFA and no stroma conditions. Furthermore, the same pattern was also observed for RPMI-8226 and U266 cells in the context of MM primary BMSCs (Additional file 6: Figure S6A-D). GFP-based cytotoxicity assays also showed that BMSCs induced resistance to BTZ and CFZ (Additional file 5: Figure S5D and Additional file 6: Figure S6E). The above observations indicate that direct cell-cell adhesion is more effective in promoting drug resistance in MM cells than soluble factors released from BMSCs.

### BMSCs modulate an array of miRNAs in MM cells in the presence of BTZ

**Fig. 3:**
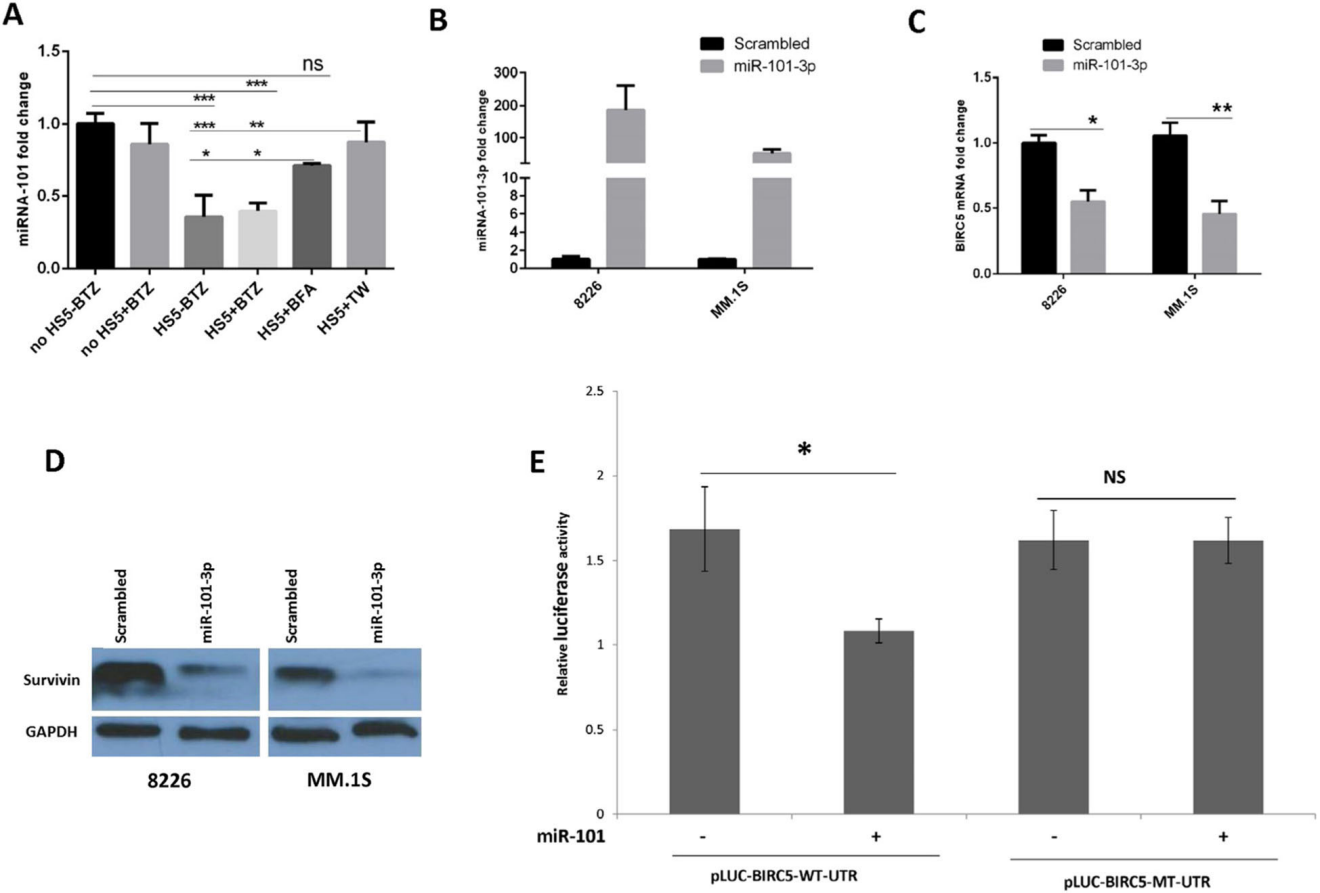
BMSCs suppress miR-101-3p which directly targets BIRC5. **a** MM.1S cells were cultured for 24 h in the following settings: cultured alone with or without 5 nM BTZ, co-cultured with HS-5 cells with or without 5 nM BTZ, co-cultured with HS-5 cells pre-treated with BFA or separated with a TW insert. Cells from all conditions were harvested, cDNAs isolated and used in real time PCR for analysis of BIRC5 mRNA expression. Analyzed data are from two separate experiments, *p < 0.05, **p < 0.01, ***p < 0.001. **b** MM cells were transduced with viral particles of miR-101-3p or negative control, harvested after puromycin selection of eGFP^+^ population to be examined in real time PCR for confirmation of transduction. **c** MM cells with overexpressed miR-101-3p were harvested and the cDNAs were applied to real time PCR for measuring BIRC5 mRNA level. **d** MM cells with overexpressed miR-101-3p were harvested and lysates were used in WB to detect survivin protein. **e** The HEK-293 T cells were transiently co-transfected with the mutant or wild type BIRC5-UTR luciferase reporter vectors together with miR-101 expressing or control vectors. Relative luciferase activities were analyzed 48 h after transfection

Having shown that BMSCs upregulate survivin in MM cells, we sought to explore whether this upregulation of survivin is associated with miRNAs. To this end, we co-cultured MM.1S cells with HS-5 cells and in parallel with MM.1S cells alone were incubated for 24 h in the presence of BTZ. MM cells were harvested, and isolated miRNAs were applied to qPCR array (Apoptosis Pathway, 84 miRNAs). The miRNA qPCR array showed that BMSCs modulated multiple miRNAs in MM cells, with some up-and some down-regulated (Additional file 7: Figure S7, clusterogram). While it is impossible to exclude the role of other miRNAs in MM stroma-mediated drug resistance, we selected miR-101-3p because it showed a high level of downregulation in the array (0.0327 or around 30 folds), and its role in stroma-induced drug resistance has not been explored in MM. The other highly downregulated miRNA in the array was miR-29b, however; it has been extensively studied in MM [16, 33] and also shown to be regulated to some level by BMSCs in MM [34]. As shown in Fig. 3a and Additional file 8: Figure S8, miR-101-3p was suppressed in MM.1S and RPMI-8226 cells by BMSCs, however; this effect was reversed partially by BFA but more significantly by TW inserts. In addition, BTZ did not affect miR-101-3p level indicating specific stroma-mediated downregulation of this miRNA (Fig. 3a). These results indicate that direct cell-cell adhesion is more efficient in stroma-mediated downregulation of miR-101-3p in MM cells.

### Survivin is directly targeted by miR-101-3p

As miR-101-3p and survivin were oppositely modulated by BMSCs; we attempted to understand if survivin could be a target of this miRNA. Using miRTarBase we found 3 binding sites of miR-101-3p seed sequence in 3’UTR of survivin (Additional file 9: Figure S9). To investigate whether miR-101-3p targets BIRC5, we first overexpressed this miRNA in RPMI-8226 and MM.1S cell lines, which was confirmed in qPCR (Fig. 3b). Overexpression of miR-101-3p significantly suppressed BIRC5 mRNA (Fig. 3c) and protein (Fig. 3d). Luciferase assays also showed that luciferase activity significantly decreased in the presence of wild-type BIRC5 3’UTR clone and miR-101-3p, while it did not happen in the presence of mutant 3’UTR clone (Fig. 3e). On the other hand, miR-101-3p level in survivin-silenced cells did not change (data not shown).

### miR-101-3p - BIRC5 axis regulates MM cells’ viability in stroma context

**Fig. 4:**
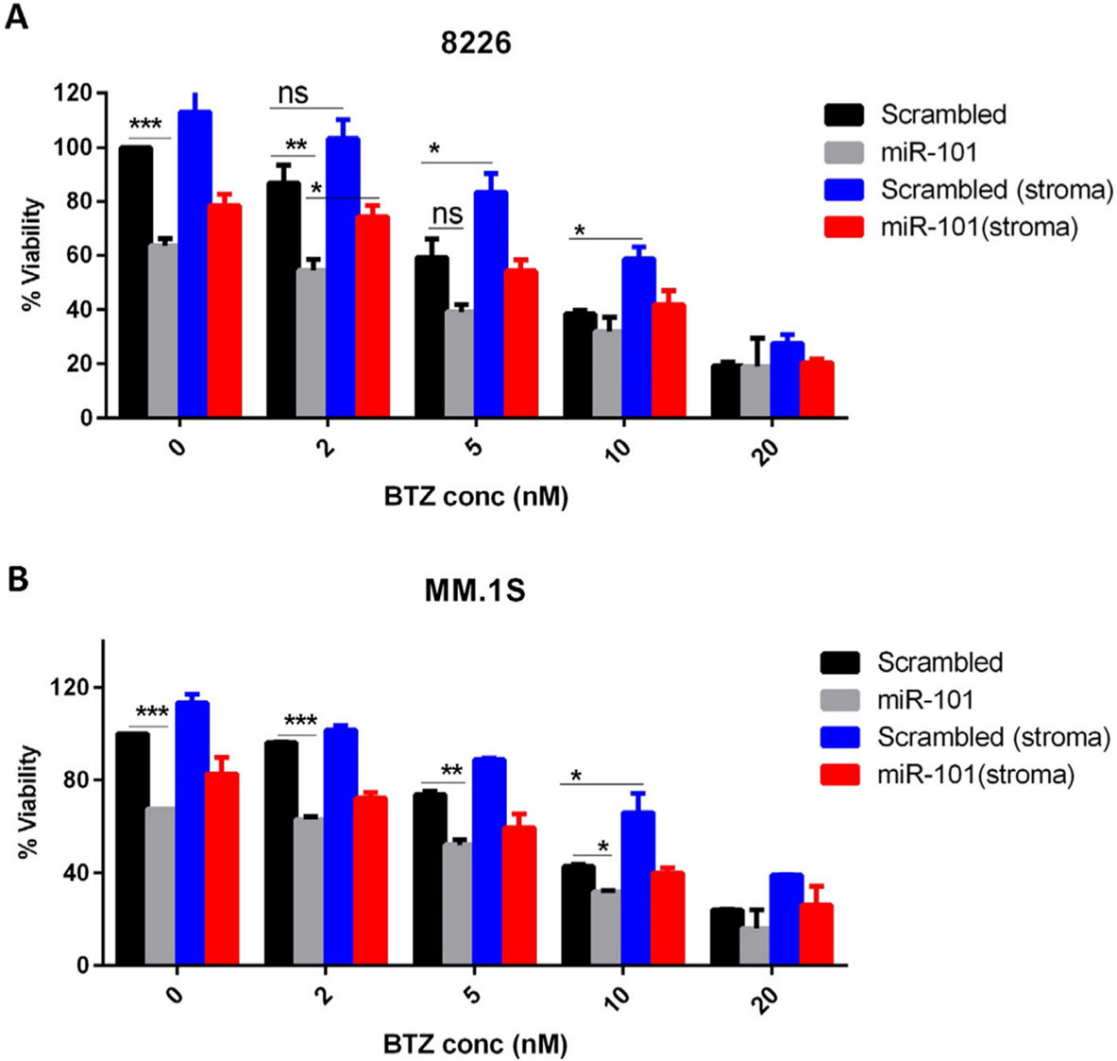
MiR-101-3p overexpression triggers cell death and partially overcomes stroma-mediated drug resistance. **a,b** miR-101-3p or miR-NC transduced RPMI-8226 and MM.1S cells were exposed to different concentrations of BTZ in the presence or absence of HS-5 cells and cytotoxicity of the drug was evaluated using GFP-based cytotoxicity assay as explained in the M&M. Graph shows data analysis from three separate experiments, *p < 0.05**,** **p < 0.01, ***p < 0.001

**Fig. 5:**
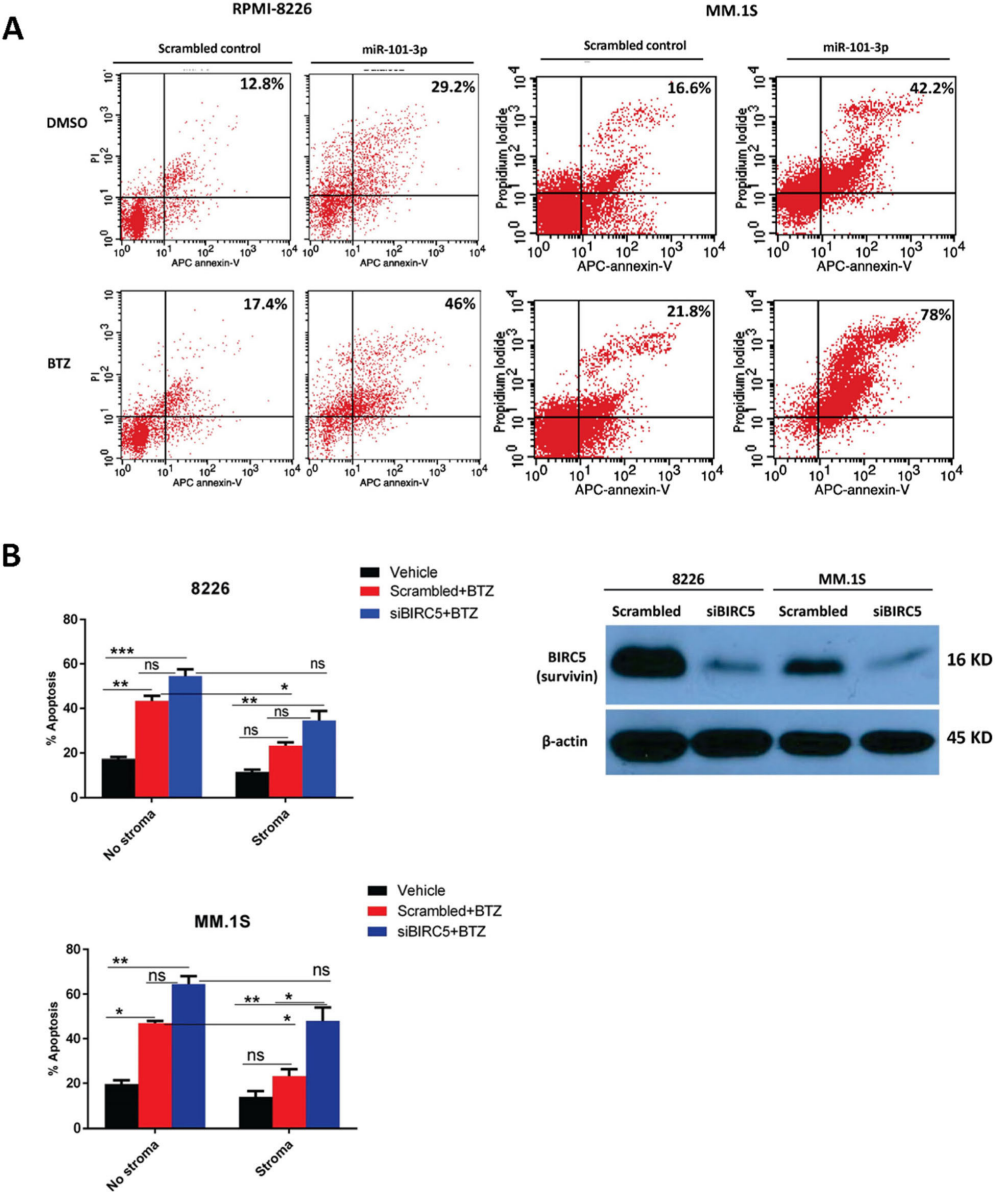
miR-101-3p overexpression or BIRC5 silencing triggers apoptosis of MM cells and partially overcomes stroma-induced BTZ resistance. **a** miR-101-3p or miR-NC transduced RPMI-8226 and MM.1S cells were treated with 5 nM BTZ for 24 h and percent apoptosis was determined using APC-annexin V/PI staining in flow cytometry. Figures are representatives of two separate experiments. **b** MM cells were transfected with BIRC5 siRNA using Lipofectamine RNAiMAX or scrambled control. Twenty four hours post-transfection, samples were treated with 5 nM BTZ in the presence or absence of HS-5 cells and incubation extended to another 24 h. Cells were then harvested for annexin-V/PI apoptosis analysis in flow cytometry and immunoblot detection of survivin protein. Graphs show the data analysis from two separate experiments, *p < 0.05**,** **p < 0.01, ***p < 0.001

**Fig. 6:**
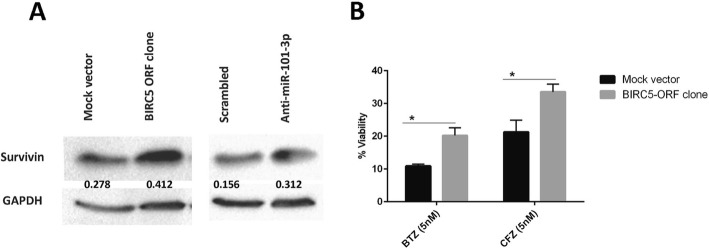
miR-101-3p/BIRC5 axis regulates MM cell viability in BTZ context. **a,b** RPMI-8226 cells were transfected transiently with cDNA ORF clone of human BIRC5 or the empty vector using lipofectamine 3000. Cells were washed 72 h after transfection, incubated with 5 nM BTZ or CFZ for 48 h and viability was examined in MTT assay (**b**). The same cells were also transfected in parallel with synthetic inhibitor of miR-101-3p or the scrambled control. Cell lysate was prepared 48 h after transfection and applied to WB for measuring survivin protein level. For these experiments, signals from the WB bands were captured using the ChemiDoc XRS+ System (Bio-Rad). For densitometric quantification of WB bands, we used ImageJ software. The densities of survivin bands were divided by densities of their relevant actin bands and reported as shown

Wealth of evidence support the crucial role of miRNAs in regulation of cancer cell death [35]. To explore whether miR-101-3p is required for stroma-induced drug resistance in MM, we evaluated cytotoxic and apoptotic response of miR-101-3p transduced MM cells to BTZ in the presence of BMSCs. Overexpression of miR-101-3p in RPMI-8226 and MM.1S cells not only triggered cell death but also increased BTZ-induced toxic effects in the presence of BMSCs partially overcoming drug resistance (Fig. 4a,b). In addition, miR-101-3p induced apoptosis in above cells and greatly sensitized them to BTZ in the absence of stroma (Fig. 5a). In other experiments to confirm whether survivin as the target also contributes to stroma-induced drug resistance, BIRC5 mRNA was silenced using siRNA and MM cell viability was assessed with flow cytometry staining. As shown in Fig. 5b, silencing efficiently reduced the survivin protein which at functional level led to sensitization of MM cells to BTZ in the presence of BMSCs partially overcoming stroma-mediated drug resistance. On the other hand, overexpression of BIRC5 using transient transfection of RPMI-8226 cells with an open reading frame (ORF) cDNA clone significantly enhanced their resistance to BTZ confirming the important regulatory function of BIRC5 in MM cell viability (Fig. 6a,b). Inhibition of tumor suppressor miRNAs has been shown to favor cell growth and viability [36, 37]. To elucidate whether miR-101-3p indeed regulates BIRC5 expression, RPMI-8226 cells were transiently transfected with synthetic inhibitor of miR-101-3p and were found to moderately upregulate survivin protein (Fig. 6a). These findings indicate that BMSCs may engage miR-101-3p/BIRC5 axis as a regulatory system to control viability and drug response of MM cells.

## Discussion

It is well established that genetic changes per se cannot be the only drivers of proliferation, survival and drug resistance of MM cells but close interaction of these cells with bone marrow microenvironment components especially BMSCs also plays a critical role [38, 39]. Here, we first show that HS-5 cells and MM patient-derived BMSCs induce resistance to BTZ and CFZ in MM cells mostly through direct cell-cell adhesion. Next, to investigate the changes at gene expression level in MM cells, and also to understand the mechanism of this resistance at molecular level, we performed mRNA and miRNA qPCR arrays on MM cells co-cultured with BMSCs and compared with MM cells cultured alone, with BTZ present in all conditions. It turned out that BMSCs upregulated some oncogenic and anti-apoptotic targets including MCL-1, c-MYC and BIRC5 (survivin) among others. However, for downstream exploration we focused on survivin, because it was not affected by BTZ in stroma context while c-MYC and MCL-1 were suppressed by the drug in the presence of stroma.

To the best of our knowledge, our study is the first to demonstrate that with BTZ added upfront to BMSCs-MM cell co-cultures, BMSCs downregulate miR-101-3p in MM cells which directly targets survivin and neither this miRNA nor survivin is affected by BTZ in the presence of stroma. Additionally, ectopic expression of survivin rendered MM cells resistant to BTZ and inhibition of miR-101-3p enhanced survivin protein level supporting the notion that miR-101-3p is a potential regulator of survivin hence drug response of MM cells. These findings suggest that BMSCs may exploit miR-101-3p/survivin axis as a general mechanism to protect MM cells against anti-myeloma drugs and further pinpoint the therapeutic targeting of this axis to overcome stroma-mediated drug resistance in MM. It should also be mentioned that role of other miRNAs in regulation of survivin cannot be ignored. For instance, miR-34a, miR-203, miR-16 and miR-218 which were included in our array are among the miRNAs reported to regulate survivin expression [40]. However; in our system, miR-34a and miR-218 did not change, miR-16 was downregulated but much less than miR-101-3p and miR-203 was upregulated. Using BFA in BMSCs or a TW insert between BMSCs and MM cells, we found that direct cell-cell adhesion contributed largely to stroma-induced upregulation of survivin, suppression of miR-101-3p and resistance to drugs. Consistent with our result on survivin, another study found that survivin was upregulated in MM cell lines by mesenchymal stromal cells mostly through direct cell-cell adhesion [41], however; the authors did not explore the role of miRNAs. It is important to mention that further in-depth exploration of the mechanisms underlying BMSC-mediated modulation of miR-101-3p/survivin axis in MM cells was beyond the scope of our study. However; for future studies it would be interesting to investigate whether activation of integrin and cytokine signaling pathways (e.g. ILK, JAK/STATs) following BMSC-MM interaction is linked to BIRC5 upregulation and processing / maturation of miR-101-3p. Moreover, role of miRNA-carrying exosomes derived from BMSCs will be worth exploring.

Stroma-induced resistance to BTZ in MM cells has been shown in some studies [18, 42–44]. For instance, Bar-Natan et al. demonstrated almost 100% viability of RPMI-8226 cells in the presence of HS-5 cells after 48 h incubation with 10 nM BTZ. BMSCs induced about 50% resistance to BTZ (2 nM, 48 h) in MM cells as reported by Sripayap et al. However; in these studies, possible involvement of miRNAs which was the important aspect of our research, was not investigated. In functional analyses, we observed that ectopic expression of miR-101-3p or BIRC5 silencing triggered cell death in MM cells and sensitized them to BTZ in the presence of BMSCs overcoming stroma-mediated drug resistance. Importantly, our findings on apoptotic effects of miR-101-3p overexpression in MM cells are in line with in vivo anti-tumor activity of miR-101 mimics in hepatocellular carcinoma xenografts [45]. Furthermore, BIRC5 upregulation in MM patients under BTZ therapy was linked to BTZ resistance, disease progression and worse progression-free survival [46], survivin inhibitor (YM155) also displayed significant anti-myeloma activity in MM xenograft model [29]. These studies support our findings that targeting of survivin/miR-101-3p axis has therapeutic potential in MM.

## Conclusions

Our data provide new insight into the role of miRNAs in bone marrow stroma-mediated drug resistance by identifying miR-101-3p-survivin interaction as a novel mechanism regulating such resistance. Our study suggests miR-101-3p-survivin axis as a potential druggable target to overcome stroma-induced drug resistance in MM and provides framework to develop more efficient anti-myeloma therapies.

## Supplementary information

**Additional file 1: Figure S1.** Clusterogram of the qPCR-based mRNA array (84 genes) for U266 cell line is shown here as representative, details in the M&M.

**Additional file 2: Figure S2.** The graph for U266 cell line is the re-analysis of selected genes from an array of 84 genes. The graph shows the fold changes of transcripts in MM cells co-cultured with HS-5 cells compared to MM cells cultured alone.

**Additional file 3: Figure S3.** Modulation of MCL and c-MYC proteins in RPMI-8226 cells by HS-5 cells with and without BTZ.

**Additional file 4: Figure S4.** Analysis of GSE31159 dataset from GEO database related to 13 MM primary tumor samples. The graph shows the fold changes of BIRC5 mRNA in patient malignant plasma cells co-cultured with HS-5 cells relative to the cells cultured alone.

**Additional file 5: Figure S5.** BMSCs induce resistance to BTZ in MM cells mostly through direct cell-cell adhesion. **(A-C)** GFP-tagged MM cell lines RPMI-8226, MM.1S and U266 were cultured alone or cultured with HS-5 cells in 3 settings: seeded on HS-5 cells, separated from HS-5 cells using a TW insert, or seeded on HS-5 cells pre-treated with BFA. All conditions were treated with 5 nM BTZ for 48 h. Mono and co-cultures with no BTZ were used as controls. Percent apoptosis of gated GFP+ cells was determined with APC-annexin V/PI FACS analysis. Bar graphs are data analyses from two separate experiments, *p < 0.05, **p < 0.01, ***p < 0.001. (**D**) Primary MM BMSCs induce resistance to cytotoxic effects of BTZ in MM cells. GFP-tagged HMCLs in phenol red-free RPMI+FBS were seeded on patient-derived BMSC-coated wells, after 6 h different concentrations of drugs were added. GFP fluorescent intensity was measured through a fluorescence plate reader after 72 h.

**Additional file 6: Figure S6.** BMSCs induce resistance to CFZ in MM cells mainly through cell-cell adhesion. (**A**) GFP-tagged RPMI-8226, MM.1S or U266 cells were cultured alone or with HS-5 cells in 2 settings: seeded on HS-5 cells or seeded on HS-5 cells pre-treated with BFA. All conditions were treated with 5 nM CFZ for 48 h. Mono and co-cultures with no CFZ were used as controls. Percent apoptosis of gated GFP+ cells was determined with APC-annexin V/PI FACS analysis. (**B-D**) GFP-tagged RPMI-8226 or U266 cells were cultured alone or with MM primary BMSCs in 3 settings: seeded on primary BMSCs, separated from primary BMSCs using a TW insert, or seeded on primary BMSCs pre-treated with BFA. All conditions were treated with 5 nM CFZ for 48 h. Mono and co-cultures with no CFZ were used as controls. Percent apoptosis of gated GFP+ cells was determined using APC-annexin V/PI FACS analysis. (**E**) HS-5 cells protect MM cells against cytotoxic effects of BTZ and CFZ. GFP-tagged HMCLs in phenol red-free RPMI+FBS were seeded on HS-5-coated wells in a 96-well plate, after 6 h different concentrations of drugs were added. GFP fluorescent intensity was measured through a fluorescence plate reader after 72 h.

**Additional file 7: Figure S7.** Clusterogram of the qPCR-based miRNA array (84 miRNAs) for MM.1S cell line, details of the procedure in the M&M.

**Additional file 8: Figure S8.** BMSCs suppress miR-101-3p in MM cells. RPMI-8226 cells were cultured for 24 h in the following settings: cultured alone, co-cultured with HS-5 cells, co-cultured with HS-5 cells pre-treated with BFA or separated with a TW insert. Cells from all conditions were harvested, cDNAs isolated and used in real time PCR for analysis of miR-101-3p expression. Data are from technical triplicates in one experiment, *p < 0.05, **p < 0.01, ***p < 0.001.

**Additional file 9: Figure S9.** Three binding sites in 3’UTR of BIRC5 for miR-101-3p seed sequence using online bioinformatics resources (miRTarBase).

**Additional file 10.** Supplementary methods. Detailed description of luciferase reporter assays for miR-101/BIRC5 3’UTR binding (wild-type and mutant clones) and western blotting.

## Data Availability

All data generated or analyzed during this study are included in this published article.

## Abbreviations

BCL2: B-cell lymphoma 2
BFA: Brefeldin-A
BIRC5: Baculoviral inhibitor of apoptosis repeat-containing 5
BMMCs: Bone marrow mononuclear cells
BMSCs: Bone marrow stromal cells
BTZ: Bortezomib
CAMDR: Cell adhesion-mediated drug resistance
CDK1: Cyclin-dependent kinase 1
CFZ: Carfilzomib
FOS: FBJ Murine Osteosarcoma Viral Oncogene Homolog
HMCLs: Human myeloma cell lines
ILK: Integrin-linked kinase
JAK/STAT: Janus kinase / signal transducer and activator of transcription
MCL1: Myeloid cell leukemia 1
MM: Multiple myeloma
SP1: Specificity protein 1
TW: Transwell
