# Supplementary figures and images for: Ectopic expression of BIRC5-targeting miR-101-3p overcomes bone marrow stroma-mediated drug resistance in multiple myeloma cells
Source: BMC Cancer. 2019 Oct 21;19:975. doi: 10.1186/s12885-019-6151-x (PMC6805455; doi:10.1186/s12885-019-6151-x)

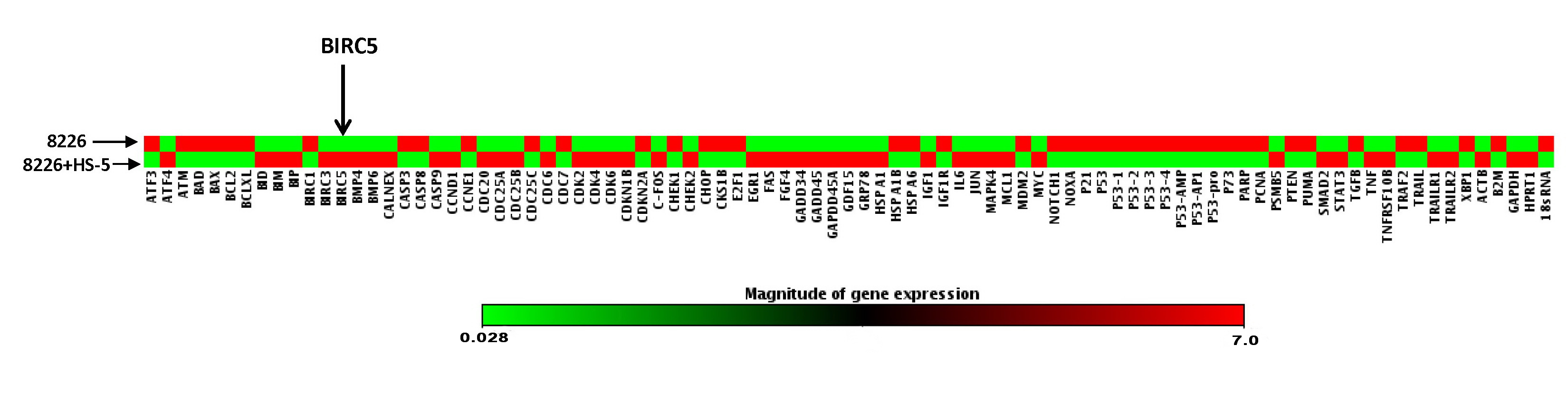

Supplement: Supplementary file 1 — Additional file 1: Figure S1. Clusterogram of the qPCR-based mRNA array (84 genes) for U266 cell line is shown here as representative, details in the M&M. [file 12885_2019_6151_MOESM1_ESM.jpg]

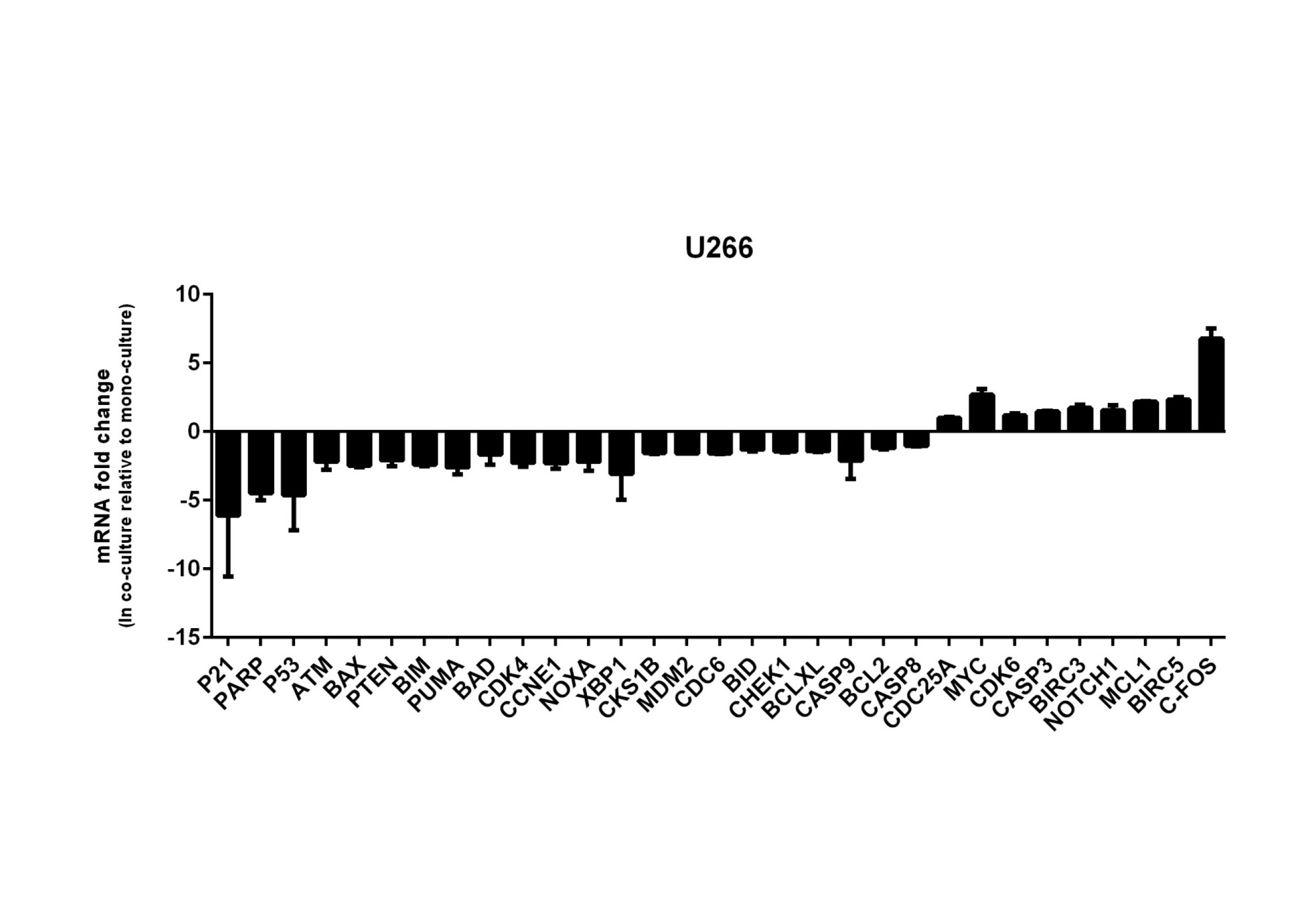

Supplement: Supplementary file 2 — Additional file 2: Figure S2. The graph for U266 cell line is the re-analysis of selected genes from an array of 84 genes. The graph shows the fold changes of transcripts in MM cells co-cultured with HS-5 cells compared to MM cells cultured alone. [file 12885_2019_6151_MOESM2_ESM.jpg]

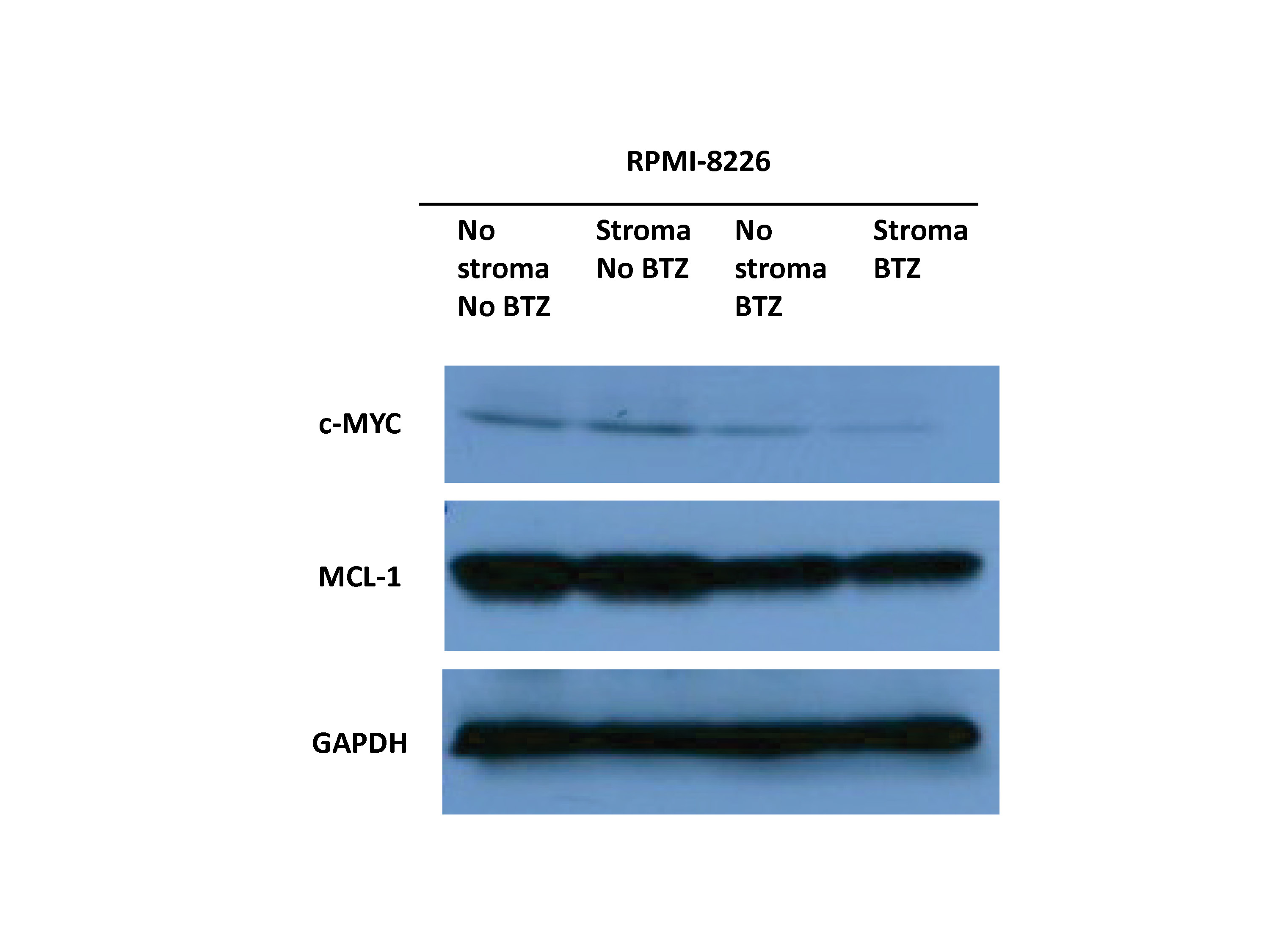

Supplement: Supplementary file 3 — Additional file 3: Figure S3. Modulation of MCL and c-MYC proteins in RPMI-8226 cells by HS-5 cells with and without BTZ. [file 12885_2019_6151_MOESM3_ESM.jpg]

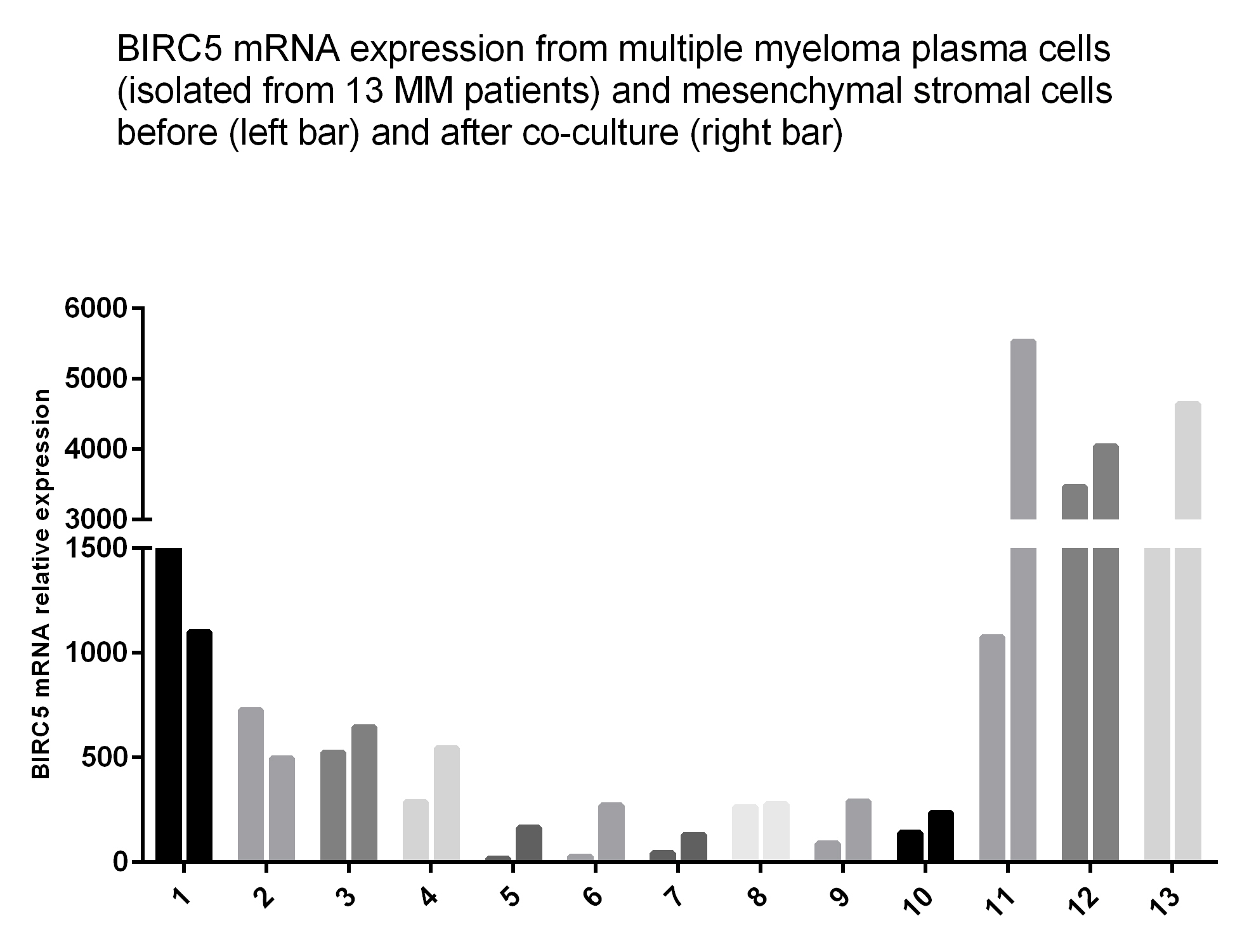

Supplement: Supplementary file 4 — Additional file 4: Figure S4. Analysis of GSE31159 dataset from GEO database related to 13 MM primary tumor samples. The graph shows the fold changes of BIRC5 mRNA in patient malignant plasma cells co-cultured with HS-5 cells relative to the cells cultured alone. [file 12885_2019_6151_MOESM4_ESM.jpg]

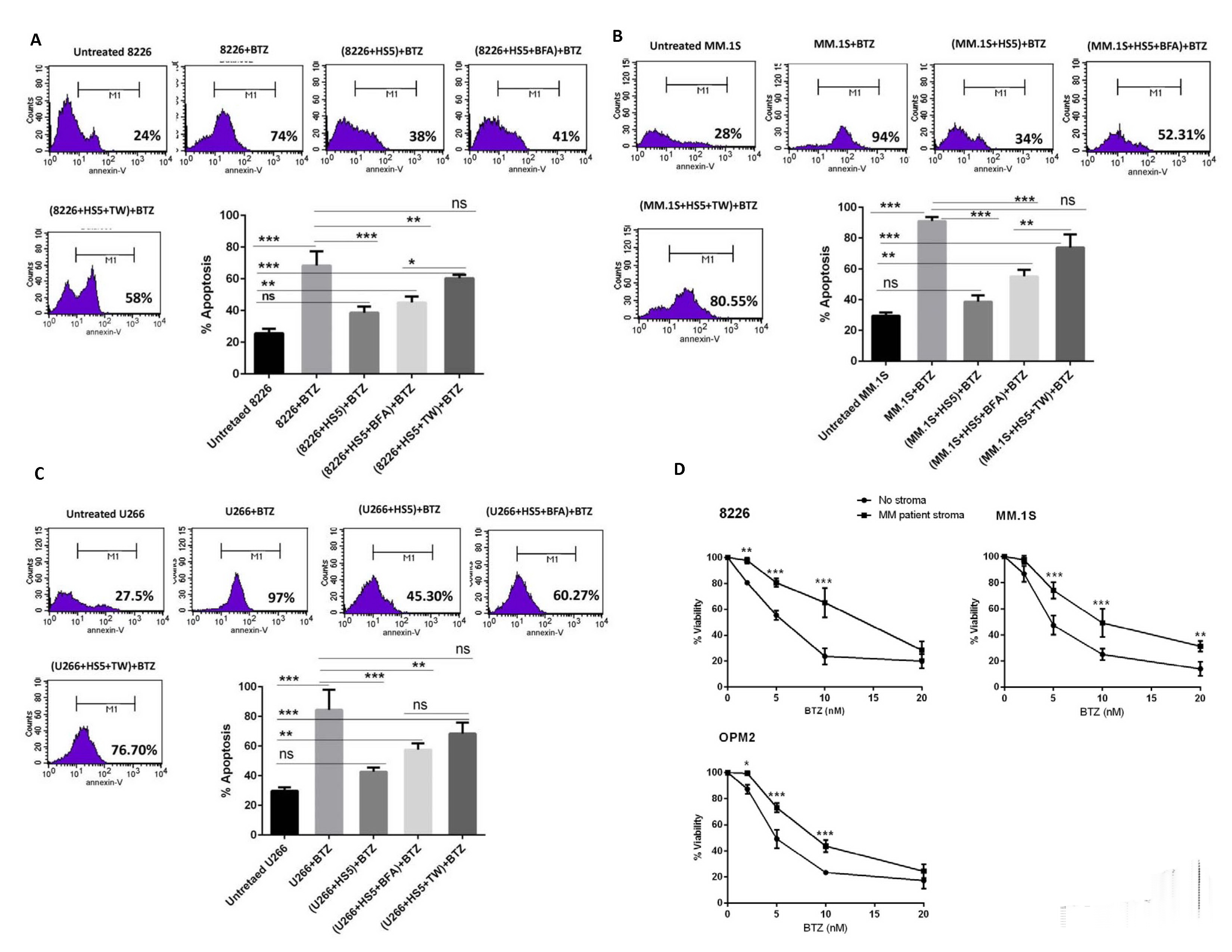

Supplement: Supplementary file 5 — Additional file 5: Figure S5. BMSCs induce resistance to BTZ in MM cells mostly through direct cell-cell adhesion. (A-C) GFP-tagged MM cell lines RPMI-8226, MM.1S and U266 were cultured alone or cultured with HS-5 cells in 3 settings: seeded on HS-5 cells, separated from HS-5 cells using a TW insert, or seeded on HS-5 cells pre-treated with BFA. All conditions were treated with 5 nM BTZ for 48 h. Mono and co-cultures with no BTZ were used as controls. Percent apoptosis of gated GFP+ cells was determined with APC-annexin V/PI FACS analysis. Bar graphs are data analyses from two separate experiments, *p < 0.05, **p < 0.01, ***p < 0.001. (D) Primary MM BMSCs induce resistance to cytotoxic effects of BTZ in MM cells. GFP-tagged HMCLs in phenol red-free RPMI+FBS were seeded on patient-derived BMSC-coated wells, after 6 h different concentrations of drugs were added. GFP fluorescent intensity was measured through a fluorescence plate reader after 72 h. [file 12885_2019_6151_MOESM5_ESM.jpg]

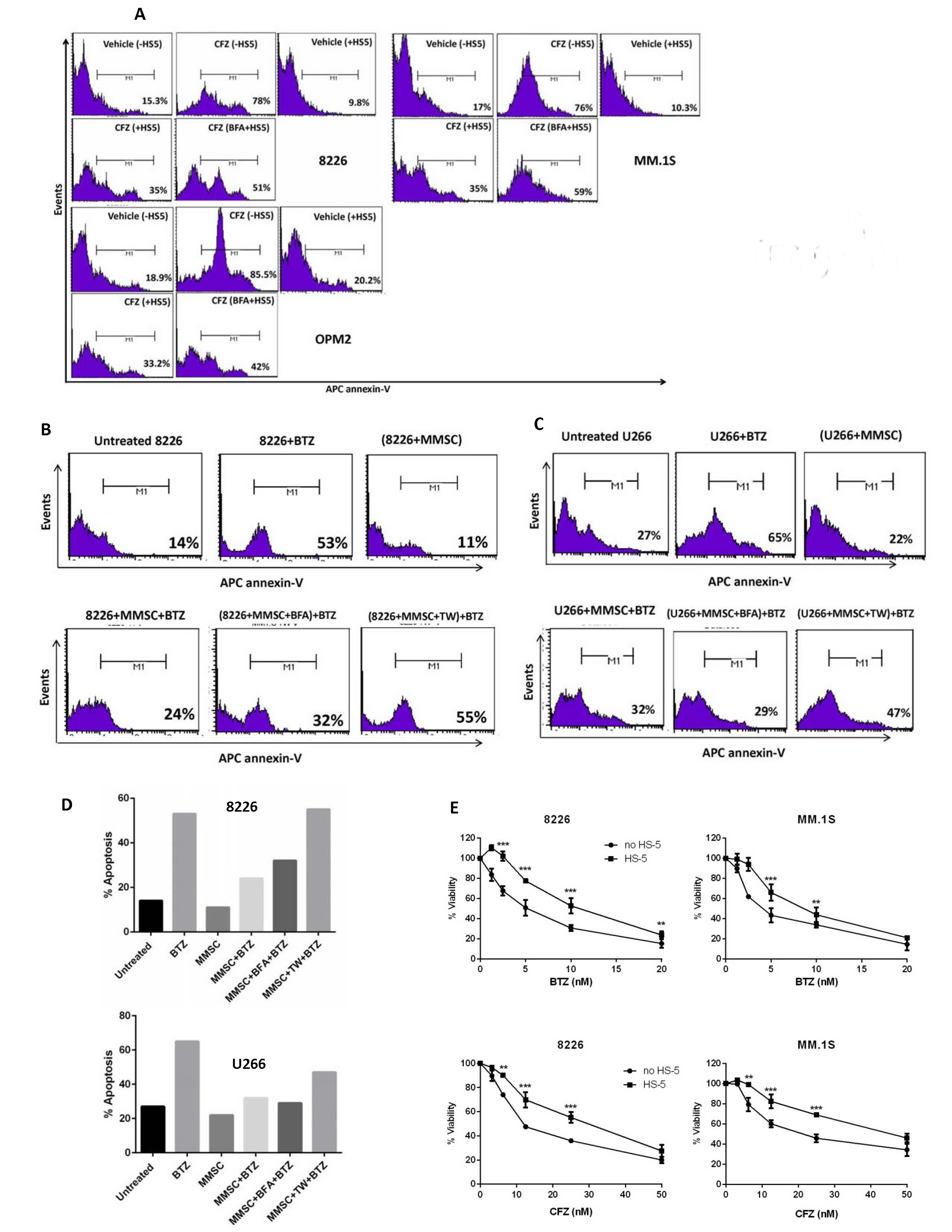

Supplement: Supplementary file 6 — Additional file 6: Figure S6. BMSCs induce resistance to CFZ in MM cells mainly through cell-cell adhesion. (A) GFP-tagged RPMI-8226, MM.1S or U266 cells were cultured alone or with HS-5 cells in 2 settings: seeded on HS-5 cells or seeded on HS-5 cells pre-treated with BFA. All conditions were treated with 5 nM CFZ for 48 h. Mono and co-cultures with no CFZ were used as controls. Percent apoptosis of gated GFP+ cells was determined with APC-annexin V/PI FACS analysis. (B-D) GFP-tagged RPMI-8226 or U266 cells were cultured alone or with MM primary BMSCs in 3 settings: seeded on primary BMSCs, separated from primary BMSCs using a TW insert, or seeded on primary BMSCs pre-treated with BFA. All conditions were treated with 5 nM CFZ for 48 h. Mono and co-cultures with no CFZ were used as controls. Percent apoptosis of gated GFP+ cells was determined using APC-annexin V/PI FACS analysis. (E) HS-5 cells protect MM cells against cytotoxic effects of BTZ and CFZ. GFP-tagged HMCLs in phenol red-free RPMI+FBS were seeded on HS-5-coated wells in a 96-well plate, after 6 h different concentrations of drugs were added. GFP fluorescent intensity was measured through a fluorescence plate reader after 72 h. [file 12885_2019_6151_MOESM6_ESM.jpg]

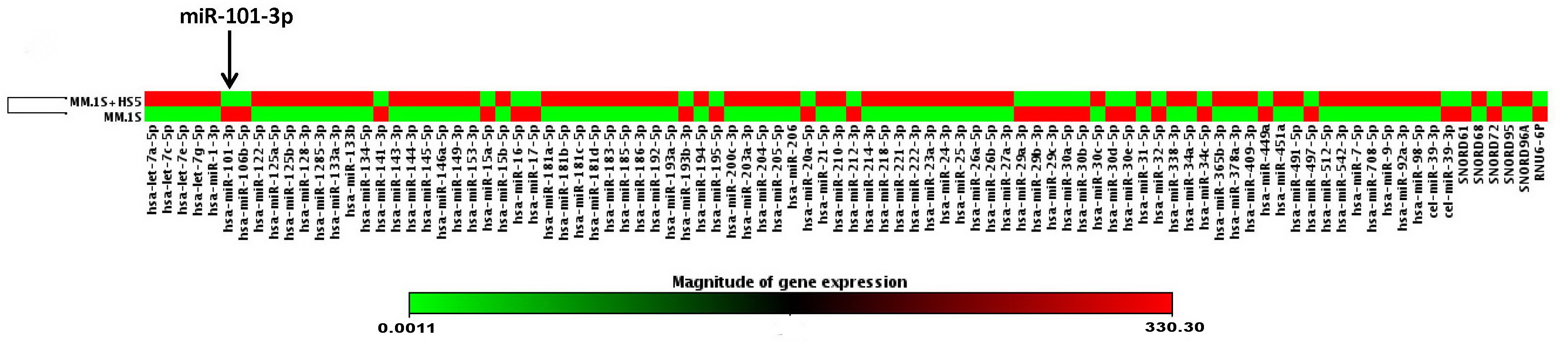

Supplement: Supplementary file 7 — Additional file 7: Figure S7. Clusterogram of the qPCR-based miRNA array (84 miRNAs) for MM.1S cell line, details of the procedure in the M&M. [file 12885_2019_6151_MOESM7_ESM.jpg]

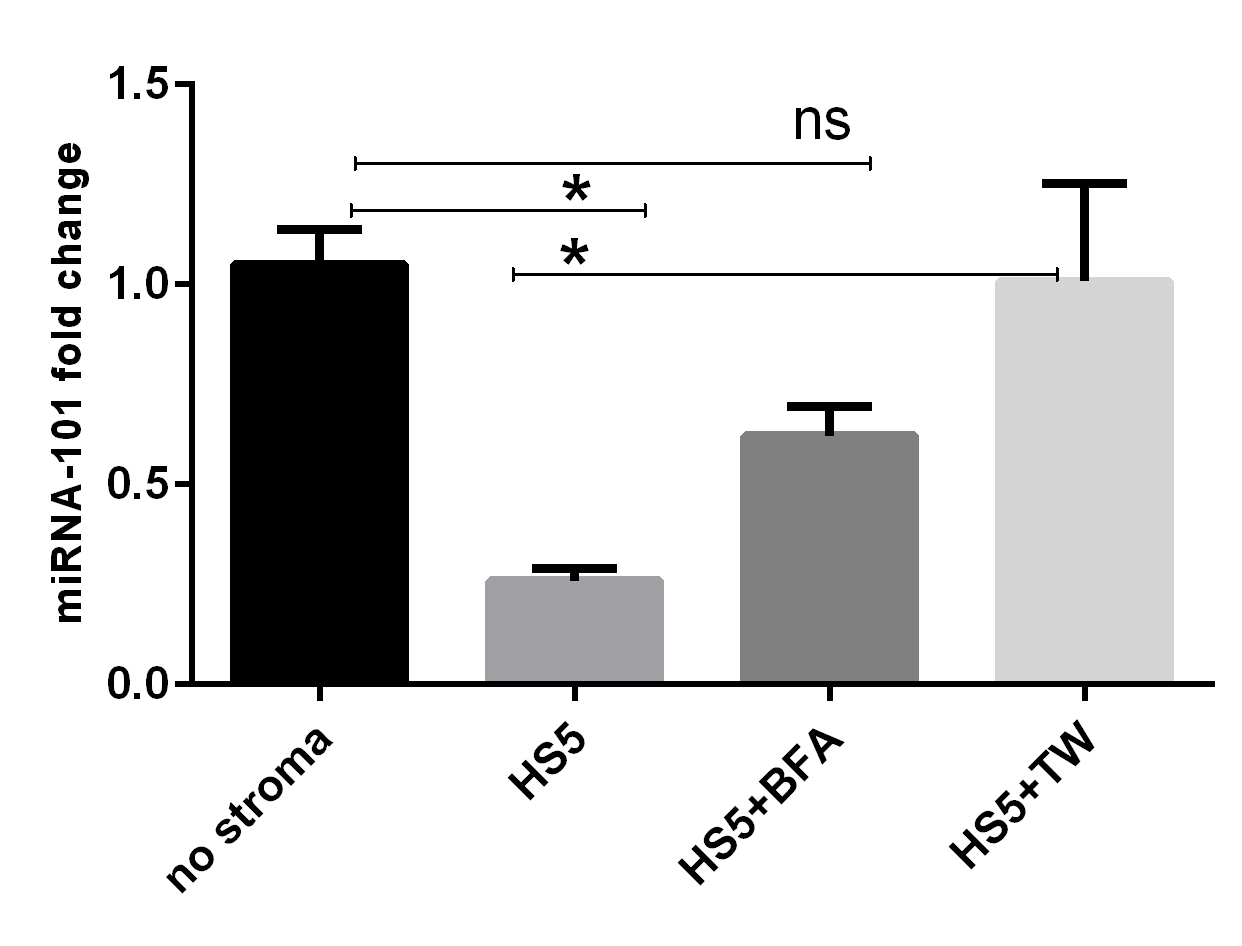

Supplement: Supplementary file 8 — Additional file 8: Figure S8. BMSCs suppress miR-101-3p in MM cells. RPMI-8226 cells were cultured for 24 h in the following settings: cultured alone, co-cultured with HS-5 cells, co-cultured with HS-5 cells pre-treated with BFA or separated with a TW insert. Cells from all conditions were harvested, cDNAs isolated and used in real time PCR for analysis of miR-101-3p expression. Data are from technical triplicates in one experiment, *p < 0.05, **p < 0.01, ***p < 0.001. [file 12885_2019_6151_MOESM8_ESM.jpg]

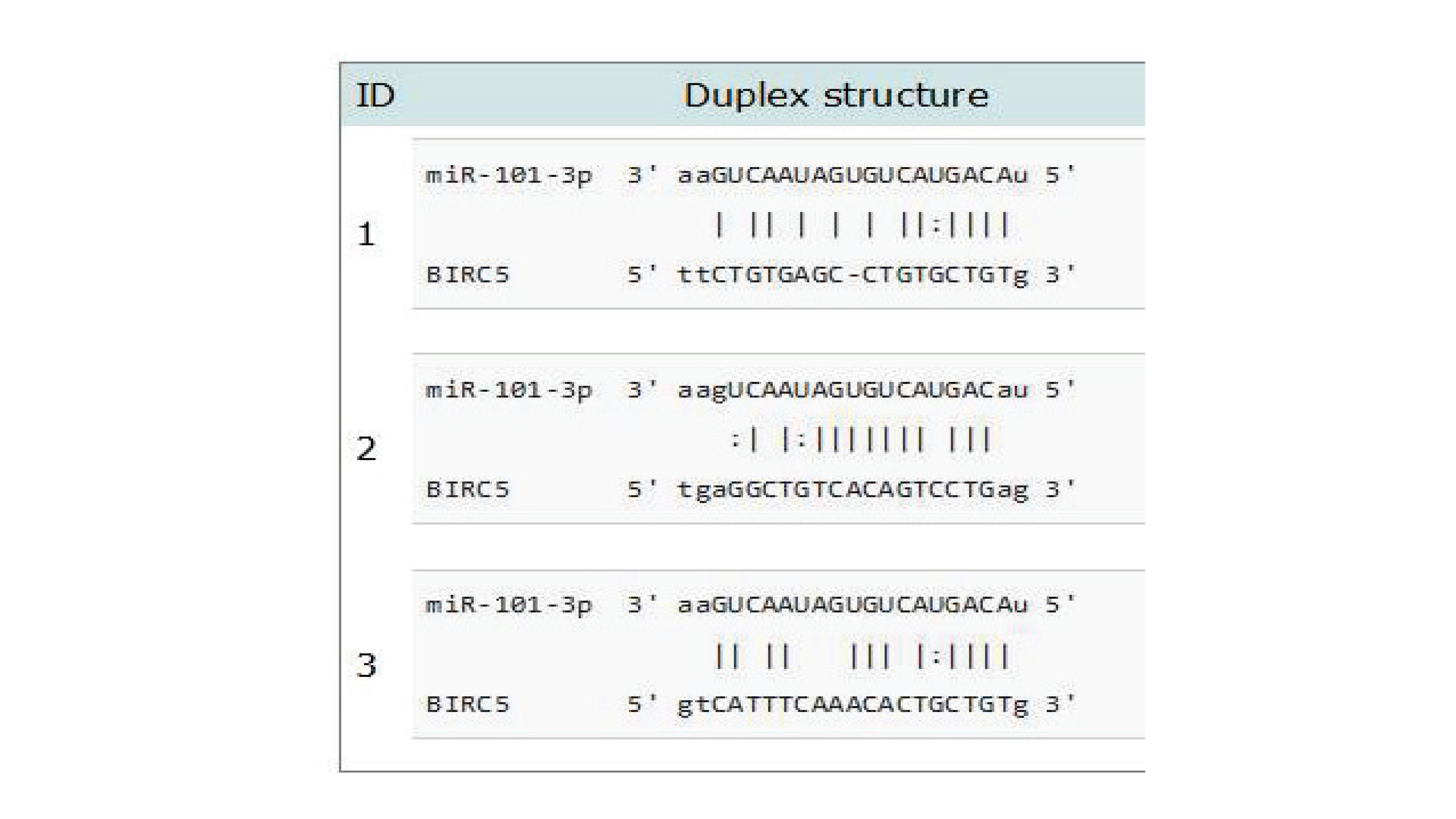

Supplement: Supplementary file 9 — Additional file 9: Figure S9. Three binding sites in 3’UTR of BIRC5 for miR-101-3p seed sequence using online bioinformatics resources (miRTarBase). [file 12885_2019_6151_MOESM9_ESM.jpg]
