# Supplementary material for: Ectopic expression of BIRC5-targeting miR-101-3p overcomes bone marrow stroma-mediated drug resistance in multiple myeloma cells
Source: BMC Cancer. 2019 Oct 21;19:975. doi: 10.1186/s12885-019-6151-x (PMC6805455; doi:10.1186/s12885-019-6151-x)
Supplement: Supplementary file 10 — Additional file 10. Supplementary methods. Detailed description of luciferase reporter assays for miR-101/BIRC5 3’UTR binding (wild-type and mutant clones) and western blotting. [file 12885_2019_6151_MOESM10_ESM.docx]

***Luciferase reporter assay***

The 3’ UTR sequence of human BRIC5 was cloned into the luciferase-expressing vector pEZX-MT01 to the downstream of the firefly luciferase gene. The mutant 3’UTR clone was constructed by introducing several mutations in miR-101-1 binding site in the 3’UTR of BIRC5 by using QuikChange Lightning Site-Directed Mutagenesis kit (Agilent Technologies Canada Inc.) according to kit manual. The 293T cells were transiently co-transfected with the mutant or wild type BIRC5-UTR luciferase reporter vectors together with miR-101 expressing or control vectors (pEZX-MR03) using lipofectamine 3000 transfection reagent. Cells were harvested 48 h after transfection for measuring *firefly* and *Renilla* relative activities using the Luc-Pair Duo-Luciferase HS Assay kit (GeneCopoeia) following the manufacture's instruction. Relative luciferase activities were analyzed as the activity of *firefly* relative to *Renilla*.

***Western blotting***

HMCLs harvested from co- or mono-cultures were washed in cold PBS and pelleted. Cell pellets were then lysed in cold RIPA buffer (150 mM NaCl, 1% Triton-X100, 0.5% sodium deoxycholate, 0.1% SDS, 50 mM Tris, pH 8.0) or another lysis buffer (150 mM NaCl, 1% Triton-X100, 50 mM Tris, pH 8.0, 10% glycerol), both containing a cocktail of protease inhibitors (Halt™ Protease Inhibitor Cocktail, ThermoFisher), and left on ice for 30 min. After spinning at 10,000×g 4°C for 15 min, the supernatants were removed and the protein concentration was measured with a BCA kit (Pierce). Thirty-fifty micrograms of total protein was applied to a 12-15% SDS gel which was then electroblotted onto a polyvinylidene fluoride (PVDF) membrane. After incubation in blocking buffer, the membranes were probed with rabbit antihuman primary antibodies (survivin, c-FOS, c-MYC, MCL-1, beta-actin or GAPDH, all from cell signaling technology) followed by specific secondary antibodies. Finally, the signals were detected using ECL (Promega) or SuperSignal West Pico Chemiluminescent (ThermoFisher) substrates if required.
